# Bone Morphogenetic Protein-2 Signaling in the Osteogenic Differentiation of Human Bone Marrow Mesenchymal Stem Cells Induced by Pulsed Electromagnetic Fields

**DOI:** 10.3390/ijms21062104

**Published:** 2020-03-19

**Authors:** Fernanda Martini, Agnese Pellati, Elisa Mazzoni, Simona Salati, Gaetano Caruso, Deyanira Contartese, Monica De Mattei

**Affiliations:** 1 Department of Medical Sciences, University of Ferrara, 44121 Ferrara, Italy; fernanda.martini@unife.it (F.M.); elisa.mazzoni@unife.it (E.M.); deyanira.contartese@unife.it (D.C.); 2 Department of Morphology, Surgery and Experimental Medicine, University of Ferrara, 44121 Ferrara, Italy; agnese.pellati@unife.it; 3 IGEA Clinical Biophysics, Carpi, 41012 Modena, Italy; s.salati@igeamedical.com; 4 Department of Biomedical and Specialty Surgical Sciences, Azienda Ospedaliero—Universitaria di Ferrara—Arcispedale Sant’Anna, University of Ferrara, 44124 Ferrara, Italy; gaetano.caruso@unife.it; 5 Laboratory Preclinical and Surgical Studies, IRCCS Istituto Ortopedico Rizzoli, 40136 Bologna, Italy

**Keywords:** BMP, osteogenic differentiation, mesenchymal stem cells, pulsed electromagnetic fields

## Abstract

Pulsed electromagnetic fields (PEMFs) are clinically used with beneficial effects in the treatment of bone fracture healing. This is due to PEMF ability to favor the osteogenic differentiation of mesenchymal stem cells (MSCs). Previous studies suggest that PEMFs enhance the osteogenic activity of bone morphogenetic protein-2 (BMP2) which is used in various therapeutic interventions. This study investigated the molecular events associated to the synergistic activity of PEMFs and BMP2 on osteogenic differentiation. To this aim, human MSCs (hMSCs) were exposed to PEMFs (75 Hz, 1.5 mT) in combination with BMP2, upon detection of the minimal dose able to induce differentiation. Changes in the expression of BMP signaling pathway genes including receptors and ligands, as well as in the phosphorylation of BMP downstream signaling proteins, such as SMAD1/5/8 and MAPK, were analyzed. Results showed the synergistic activity of PEMFs and BMP2 on osteogenic differentiation transcription factors and markers. The PEMF effects were associated to the increase in BMP2, BMP6, and BMP type I receptor gene expression, as well as SMAD1/5/8 and p38 MAPK activation. These results increase knowledge concerning the molecular events involved in PEMF stimulation showing that PEMFs favor hMSCs osteogenic differentiation by the modulation of BMP signaling components.

## 1. Introduction

Impaired fracture healing represents a major clinical problem that can lead to serious consequences and patient disability. Generally bone fractures heal by standard clinical practices, although approximately 10% patients suffer from delayed unions or non-unions [1]. Electromagnetic field (EMF) exposure represents a safe and efficient non-surgical treatment in promoting bone ununited fracture healing in clinics [2,3,4]. The use of biophysical stimulation in clinical setting is supported by excellent results of in vivo and in vitro studies, which identified the EMF effects on the cells involved in bone repair processes, especially osteoblasts and mesenchymal stem cells (MSCs) [5,6,7,8].

Specifically, EMFs have been shown to promote the differentiation of MSCs, including human MSCs (hMSCs) derived from different tissues, toward the osteoblastic lineage by increasing osteogenic transcription factor gene expression and the production of bone matrix components [7,8,9,10,11,12].

Further, it has been shown that the physical characteristics of the signal and the exposure length can influence the osteogenic effects [9,13,14]. Moreover, it has been reported that pulsed EMFs (PEMFs) increase the activity of bone morphogenetic protein-2 (BMP2), an essential growth factor for bone cells [6,7,15].

Bone morphogenetic proteins (BMPs) belong to the transforming growth factors beta (TGF-β) family, known as strong inducers of osteogenic differentiation [16]. BMP ligands signal by binding to heteromeric complexes of two types of Ser/Thr kinase receptors (BMP type I and type II receptors) [17,18]. Consequently, intra-cellular R-Smads (SMAD1/5/8) become phosphorylated and translocate into the nucleus where they cooperate with other DNA-binding proteins to regulate BMP target gene transcription including distal-less homeobox (DLX)-2/5, Runx family transcription factor 2 (RUNX2) and osterix (OSX) [19]. Further, BMPs are able to activate several non-Smad pathways involving signaling via mitogen-activated protein kinases (MAPKs) [18]. Actually, more than 20 BMPs have been identified. Among them, BMP-2, -4, -6, -7, and -9 play major roles in bone morphogenesis [16,18,20]. Notably, recombinant human BMP-2 (rhBMP2), produced by a genetically engineered Chinese hamster ovary (CHO) cell line, was the first to be introduced as a bone graft substitute and received Food and Drug Administration approval in 2002 [19]. rhBMP2 is commercially available and used in several therapeutic interventions [19]. However, following rhBMP2 use, some negative side effects have been reported probably due to the high doses applied [19,21]. Hence, there is growing interest in agents that stimulate the osteogenic differentiation of MSCs or are able to increase BMP2 activity, thus allowing lowering of its dosage. In this view, PEMFs represent a promising option because of their differentiative effects identified both in the absence and in combination with BMP2 [6,7]. However, few data are still present in literature concerning the PEMF molecular mechanisms and the involvement of specific molecular signal transduction pathways. 

The aim of the present study was to gain further knowledge into the mechanisms underlying PEMF effects on hMSCs osteogenic differentiation and the synergistic activity with BMP2; thus, the present research investigated the potential influence of PEMFs on BMP signaling. To this purpose, rhBMP2 manufactured in a CHO cell line and PEMFs with specific biophysical characteristics successfully used in previous studies were applied to stimulate osteogenic differentiation of hMSCs [7,8]. During osteogenic differentiation of hMSCs cultured in the presence of PEMF and BMP2 used alone or in combination, we investigated changes in the gene expression of BMP signaling pathway components including receptors, ligands, and nuclear target genes as well as the earlier events involved in BMP signaling, including activation of SMAD1/5/8 and MAPKs.

## 2. Results

### 2.1. Dose-Response Effects of BMP2 on hMSCs Osteogenic Differentiation

**Figure 1:**
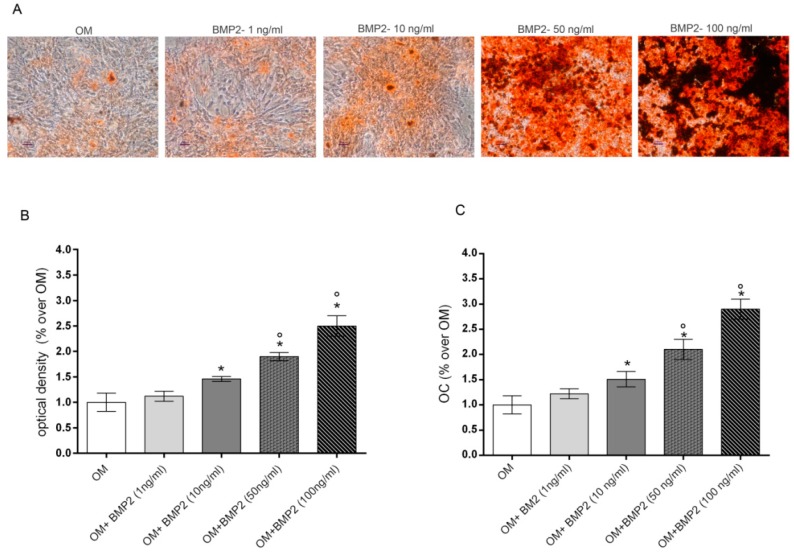
Dose-response effects of BMP2 (1, 10, 50, 100 ng/mL) on mineralization (**A**,**B**) and OC production (**C**) in hMSCs cultured in OM at 28 days. Matrix mineralization evaluated by alizarin red staining in cell monolayers (**A**) and spectrophotometrically quantified (**B**), OC levels evaluated by Elisa (**C**). *: *p* ≤ 0.05 vs. OM. °: *p* ≤ 0.05 vs. the lower dose of BMP2. Scale bar = 250 μm; Magnification = 10×.

Preliminary experiments were executed to evaluate the lowest dose of BMP2 able to stimulate osteogenic differentiation of hMSCs cultured in osteogenic medium (OM). To this aim, we evaluated the dose dependent effects induced by increasing doses of BMP2 (0–100ng/mL) in OM cultured cells on the late markers of differentiation including mineralization and osteocalcin (OC) levels at the end of differentiation in culture (28 days) [8]. Data reported in Figure 1 show a dose response effect on both mineralization, evaluated by alizarin red staining (Figure 1A,B) and OC production (Figure 1C). Specifically, the osteogenic parameters investigated were significantly increased from 10 to 100 ng/mL BMP2 in comparison to cells cultured in OM, whilst no significant effect was induced by 1 ng/mL BMP2.

### 2.2. Effects of PEMF Exposure and BMP2 on hMSCs Osteogenic Differentiation

**Figure 2:**
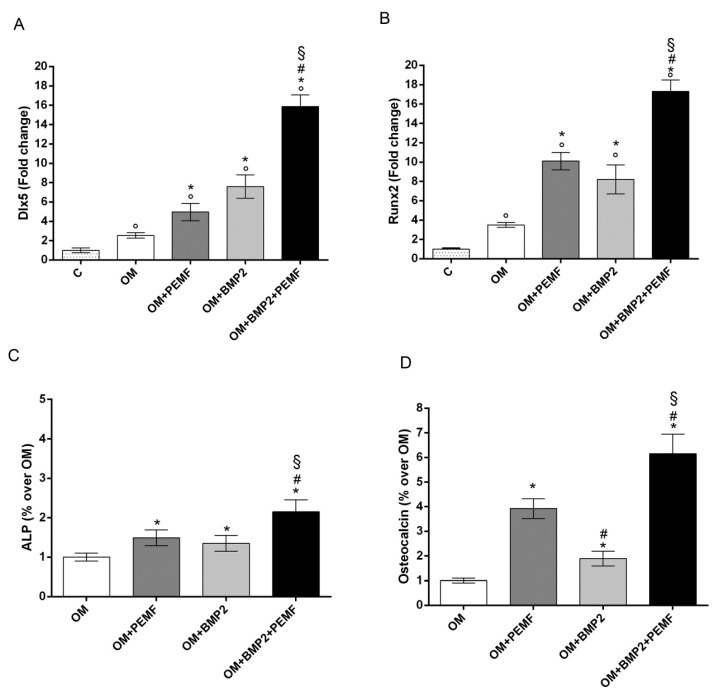
Effects of PEMFs and BMP2 (10 ng/mL) used alone or in combination on osteogenic transcription factors and biochemical markers during hMSCs osteogenic differentiation. (**A**) DLX5 and (**B**) RUNX2 gene expression by RT-qPCR at 3 days, (**C**) ALP activity at 14 days, and (**D**) OC production at 28 days. °: *p* ≤ 0.05 vs. control. *: *p* ≤ 0.05 vs. OM. #: *p* ≤ 0.05 vs. OM + PEMF. §: *p* ≤ 0.05 vs. OM + BMP2.

As 10 ng/mL was the lowest BMP2 dose able to significantly increase hMSCs differentiation, this dose was used to investigate the effects of BMP2 and PEMF treatments used alone or in combination on osteogenic differentiation. To this aim the expression of the osteogenic transcription factors DLX5 and RUNX2, alkaline phosphatase (ALP) activity, and OC production were analyzed (Figure 2). The gene expression analysis of the osteogenic transcription factors showed that both PEMF exposure and BMP2 significantly increased DLX5 and RUNX2 expression in the early phase of differentiation (day 3) (Figure 2A,B) when compared to cells cultured in OM. No significant difference between PEMF- and BMP2-induced effects were observed when each stimulus was used alone. When hMSCs cultured in OM containing BMP2 were exposed to PEMFs, a further significant increase in DLX5 and RUNX2 gene expression was observed in comparison to cells treated with PEMFs or BMP2 alone. 

Similar effects induced by PEMF exposure or BMP2 treatment were also observed when ALP activity and OC levels were analyzed (Figure 2C,D). Specifically, PEMFs stimulated ALP activity (+49%) at 14 days and OC level (+29%) at 28 days, compared to OM; BMP2 stimulated ALP activity (+35%) at 14 days and OC level (+89%) at 28 days, compared to OM. Both ALP activity and OC levels were significantly higher in cells exposed to PEMFs in the presence of BMP2, when compared to cells treated with PEMFs or BMP2 alone. 

### 2.3. Effects of PEMF Exposure and BMP2 on Gene Expression of BMPs and BMP Receptors During hMSCs Osteogenic Differentiation

**Figure 3:**
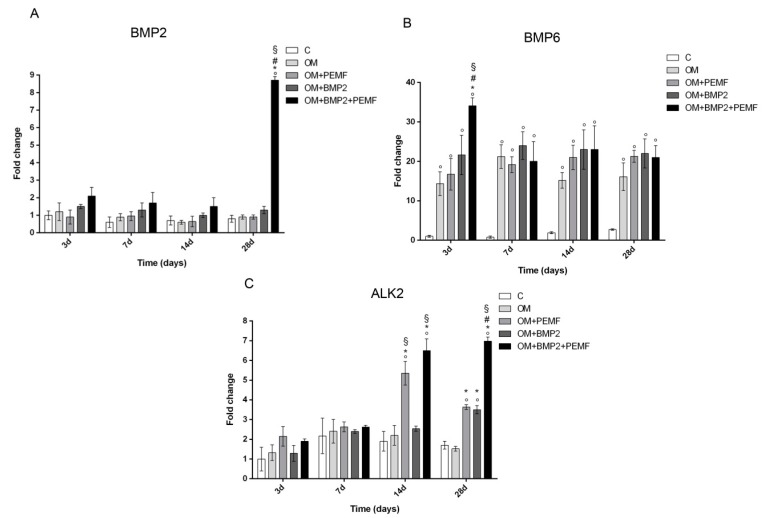
Effects of PEMFs and BMP2 (10 ng/mL) used alone or in combination on BMP2, BMP6, ALK2 gene expression, evaluated by RT-qPCR at different times points (3, 7, 14, 28 days) during hMSCs osteogenic differentiation. °: *p* ≤ 0.05 vs. control. *: *p* ≤ 0.05 vs. OM. #: *p* ≤ 0.05 vs. OM + PEMF. §: *p* ≤ 0.05 vs. OM + BMP2.

In order to investigate the potential effects of PEMF exposure on BMP signaling, we evaluated the gene expression of several components belonging to the BMP signaling pathway including BMP2, BMP6, and BMP9 which are known to induce the most potent osteogenic differentiation of MSCs and the main receptors involved in BMP signaling including BMP type I (ALK2/ACVR1, ALK3/BMPR-IA, and ALK6/BMPR-IB) and type II (BMPR-II) receptors [15,17,18,20] in cells undergoing osteogenic differentiation in all our experimental conditions. Target genes showing significant differences in their expression at any time during cell differentiation are reported in Figure 3. Among the BMPs investigated, changes in BMP2 and BMP6 gene expression were observed at selected times and culture conditions. A significant increase in BMP2 gene expression was identified at 28 days (8.7-fold) only when cells cultured in OM were treated with BMP2 and exposed to PEMFs in comparison to cells cultured in OM. BMP6 gene expression significantly increased in cells cultured in OM in comparison to control cells at all the times investigated. Further, at 3 days, when PEMFs and BMP2 were used in combination a 2.3-fold significant increase in BMP6 expression was observed in comparison to cells cultured in OM. No significant change in BMP9 gene expression was identified in all the experimental conditions, throughout the period of cell differentiation (data not shown). When we investigated potential changes in the expression of BMP receptors, we observed significant changes only in ALK2 gene expression in the middle-late phase of osteogenic differentiation. Specifically, both PEMFs and BMP2 alone enhanced ALK2 expression (PEMFs: 2.8-fold at 14 days; 2.1-fold at 28 days; BMP2: 2.05-fold at 28 days) compared to OM. Further, at 28 days in cells treated with BMP2 and exposed to PEMFs, ALK2 expression was significantly higher in comparison to cells treated with PEMFs or BMP2 alone. No significant change was observed in the expression of all the other BMP receptors analyzed at any time investigated in all the experimental conditions (data not shown).

### 2.4. Immunoblotting 

**Figure 4:**
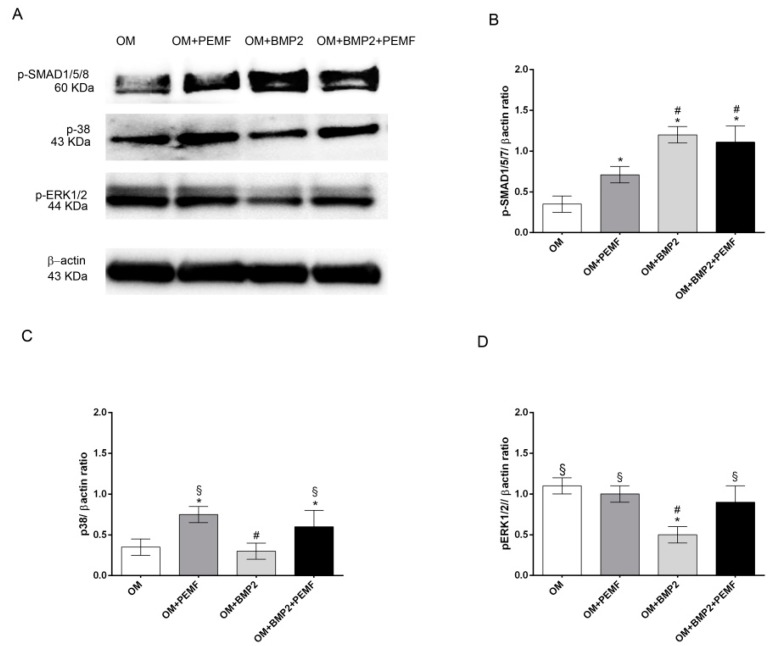
Effects of PEMFs and BMP2 (10 ng/mL) used alone or in combination on SMAD1/5/8, p38 MAPK, and ERK1/2 phosphorylation. (**A**) Cell lysates obtained in all the experimental conditions (OM, OM + PEMFs, OM + BMP2, OM + PEMFs + BMP2), electrophoresed and immunoblotted with phospho-SMAD1/5/8, phospho-p38, and phospho-ERK1/2 antibodies. β-actin antibody used to ensure equal sample loading. (**B**–**D**) Graphical representation of densitometry and Western blotting quantitative data for each protein. Results expressed as mean ± standard error of the mean of three independent experiments. *: *p* ≤ 0.05 vs. OM. #: *p* ≤ 0.05 vs. OM + PEMFs. §: *p* ≤ 0.05 vs. OM + BMP2.

To further investigate if PEMFs alone or in combination with BMP2 may modulate BMP signaling, we next analyzed the early signaling events downstream of BMP receptors including the activation by phosphorylation of SMAD1/5/8, p38 and ERK1/2 MAPKs through Western blotting analysis. Data obtained at 4 h of treatment are shown in Figure 4. All the proteins investigated appeared phosphorylated in cells cultured in OM. As expected [17,18,22], BMP2 promoted SMAD1/5/8 phosphorylation, whilst it inhibited ERK1/2 activation and did not modify p38 MAPK activation in comparison to cells cultured in OM. Differently PEMFs increased p38 MAPK phosphorylation level as well as SMAD1/5/8 activation, although to a lesser extent in comparison to BMP2 treatment. In cells treated with BMP2 and exposed to PEMFs, SMAD1/5/8 phosphorylation level did not differ from that observed in cells treated with BMP2 alone, and p38 MAPK and ERK1/2 phosphorylation levels were similar to cells treated with PEMFs alone. Similar results in protein patterns were obtained also at 24 hand the total protein amount was similar at the different time points (data not shown).

### 2.5. Effects of Dorsomorphin and SB203580 on Osteogenic Differentiation

**Figure 5:**
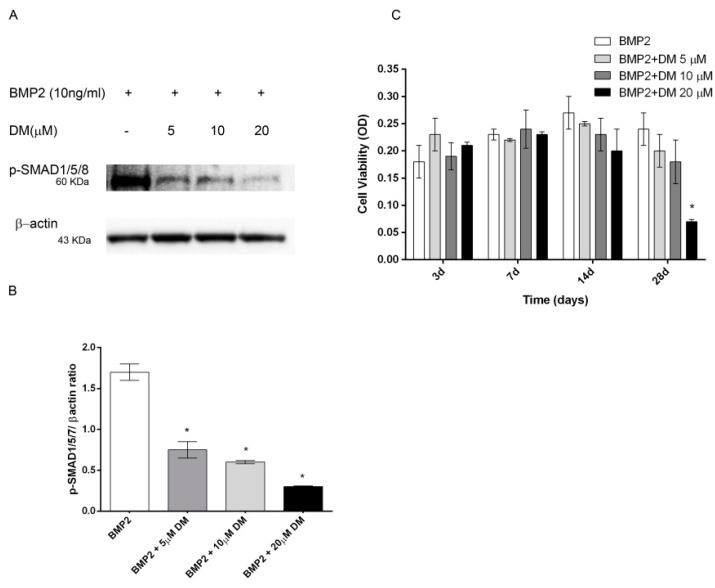
(**A**) Dose-response effects of DM on SMAD1/5/8 activation and (**B**) hMSCs viability in the presence of BMP2 (10 ng/mL). hMSCs cultured in OM + BMP2 and treated with increasing doses of DM (0, 5, 10, 20 μM). (**A**) Cell lysates electrophoresed and immunoblotted with phospho SMAD1/5/8 antibody. β-actin antibody used to ensure equivalent sample loading. (**B**) Graphical representation of densitometry and Western blotting quantitative data. (**C**) Cell viability evaluated at different time points (3, 7, 14, 28 days). *: *p* ≤ 0.05 vs. BMP2 at the corresponding time point.

**Figure 6:**
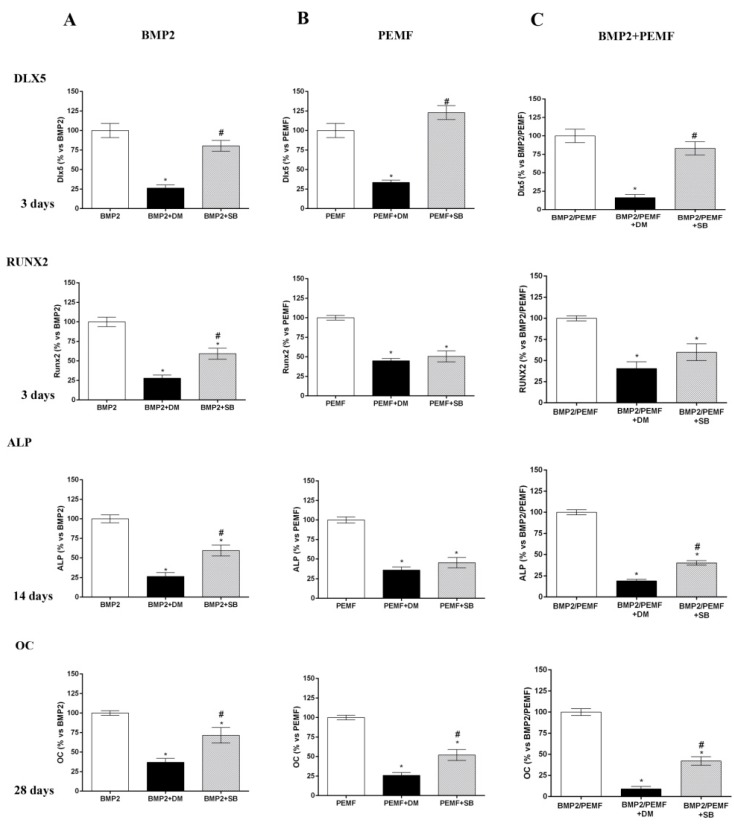
Effects of DM (5 µM) and SB203580 (10 µM) on hMSCs differentiated (**A**) in OM + BMP2, (**B**) in OM + PEMFs, (**C**) in OM + PEMFs + BMP2. DLX5 and RUNX2 gene expression (3 days), ALP activity (14 days), and OC production (28 days) evaluated. *: *p* ≤ 0.05 vs. cells differentiated in the absence of inhibitor treatment. #: *p* ≤ 0.05 vs. cells differentiated in the presence of DM.

As the main early events induced by PEMFs or BMP2 included SMAD1/5/8 and p38 MAPK phosphorylation, to further investigate the involvement of SMAD1/5/8 and p38 MAPK activation, we assessed the effects of Dorsomorphin (DM), a small BMP signaling inhibitor [23,24] and SB203580, a widely used selective inhibitor of p38 MAPK [25] on osteogenic differentiation in all our experimental conditions. To select the DM dose to use in the differentiation experiments, we firstly investigated the effects of DM at various concentrations (0, 5, 10, 20 μM) in the presence of BMP2 on both SMAD1/5/8 activation and cell viability. As shown in Figure 5A SMAD1/5/8 phosphorylation was suppressed by DM at all the doses investigated with maximal effect at 20 μM, as compared to cells treated with BMP2 alone. Data on cell viability showed that 20 μM DM treatment decreased cell viability at 28 days, whereas DM 5 or 10 μM had no detrimental effect on cell viability (Figure 5B). Based on these results, we examined the effects of 5 µM DM on cell differentiation parameters in the presence of BMP2 or PEMFs or both stimuli used in combination. Figure 6 shows the effects of DM (5 µM) and SB203580 (10 µM) on DLX5 and RUNX2 gene expression, ALP activity and OC production in hMSCs treated with PEMFs or BMP2 used alone or in combination. As shown in Figure 6, treatment with DM strongly affected osteogenic differentiation. In all the experimental conditions investigated, DM strongly inhibited DLX5 and RUNX2 gene expression, as well as ALP activity and OC production. When cells were treated with SB203580, independently from the experimental differentiative conditions, SB203580 did not significantly modify DLX5 gene expression, whilst it inhibited the other parameters investigated, although with some differences among treatments. In the presence of BMP2, the SB203580 induced inhibition on RUNX2 expression, ALP activity and OC production was lower in comparison to DM induced inhibition. In the presence of PEMFs only the SB203580 induced inhibition on OC production was lower when compared to DM inhibition. In the presence of BMP2 and PEMFs the SB203580 induced inhibition on both ALP activity and OC production was lower in comparison to DM inhibitions.

## 3. Discussion

BMP2 treatment and exposure to PEMFs characterized by specific physical parameters are used in clinics to favor bone repair in critical conditions [2,3,4,8,19,21]. Among more than 20 identified BMPs, BMP2 is considered one of the most powerful inducing osteogenesis and since 2002 it has been approved for clinical use. Although BMP2 treatment is considered as mainly safe, the need to optimize BMP2 treatments in clinics has recently arisen in order to reduce the incidence of side effects, as well as the economic costs due to the use of high BMP2 doses [19,21].

The activities of both BMP2 and PEMFs have been largely associated to their ability to enhance osteogenic differentiation [7,8,26,27,28]. Notably, some in vitro studies showed that the combination of BMP2 and PEMFs have addictive effects on hMSCs osteogenic differentiation [6,7]. 

The core purpose of this study was to investigate if PEMFs effects on osteogenic differentiation may be related to BMP signaling. Further, we aimed to verify the combined effects of PEMFs and BMP2 in the presence of the minimal dose of BMP2 able to stimulate cell differentiation in vitro. In our experiments we used rhBMP2 derived from CHO cells, differently from our previous study in which bacterial rhBMP2 was used [7] as CHO derived rhBMP2 is approved in clinics [19]. Preliminary data investigating the dose dependent effects of BMP2 showed that 10 ng/mL BMP2 was the lowest dose able to significantly increase osteogenic differentiation in our experimental conditions. Therefore, this BMP2 dose was selected to analyze the combined effects of the growth factor and PEMF exposure. In agreement with our previous study [7,8] here we show that PEMFs and BMP2 treatments, when used alone, enhance hMSCs osteogenic differentiation to the same extent as indicated by the ability to significantly increase the gene expression of the osteogenic transcription factors DLX5 and RUNX2 in the first phase of cell differentiation, as well as the middle late events of osteogenic differentiation including ALP activity and OC production. Further, at the dose of BMP2 used, we also confirmed the additive effects of PEMFs and BMP2 on all the parameters investigated. Then, in order to investigate the molecular events related to BMP signaling pathway during cell differentiation induced by BMP2, PEMFs, or both stimuli used in combination, we first analyzed the effects on the gene expression of several components of the BMP signaling pathway including other BMPs with high osteogenic potential and their receptors [15,17,18,20]. Among the BMPs investigated, the significant changes observed included a significant increase in BMP6 expression (3 days) and in BMP2 (28 days) in cells treated with BMP2 and PEMFs used in combination, compared to cells cultured in OM. Notably, BMP6 expression was also significantly increased in cells cultured in OM in the absence of BMP2 or PEMFs, in comparison to cells maintained in control medium at all the time points investigated. This result shows that the increase observed in BMP6 expression in osteogenic medium may be involved in hMSCs differentiation in agreement with the known activity of BMP6, as a potent inducer of MSCs differentiation to osteoblasts and bone formation [15,20,29,30]. Further, it suggests that the combined effects of BMP2 and PEMFs may be due at least partly to the increase in BMP6 expression. This may favor the formation of a BMP2/BMP6 heterodimer more powerful in inducing osteogenic differentiation than BMP2 or BMP6 homodimer, due to its higher affinity to BMP receptors resulting in the increased activation of BMP signaling pathways [30,31]. Therefore, the observation that PEMFs may increase BMP2/BMP6 heterodimer formation represents the basis to stimulate further investigations aimed to set-up novel protocols for treating bone defects with lower BMP2 doses. The analysis of potential changes in gene expression of BMP type I and type II receptors showed that PEMFs or BMP2 used alone or in combination could increase ALK2 expression in the middle late phase of cell differentiation. This result appears of interest because of the key role of ALK2 in the osteogenic differentiation induced by several osteogenic BMPs and suggests that PEMFs and BMP2 may act through a modulation in BMP2 or BMP6 utilization as these BMPs predominantly act by this type I receptor in hMSCs [32,33]. Further, our observation is in agreement with previous studies showing that ALK2 expression may be upregulated through BMP/SMAD signaling in MSCs [34].

When we investigated the activation of the main proteins involved in the early events of BMP signaling, Western blotting analysis showed that all the proteins investigated including SMAD1/5/8, p38, and ERK1/2 were phosphorylated in osteogenic differentiative conditions also in the absence of BMP2 and/or PEMFs. As expected [17,18,22], BMP2 further activated SMAD1/5/8, whilst PEMFs activated p38 MAPK as well as SMAD1/5/8, although to a lesser extent in comparison to the BMP2 induced activation. As we know, this is the first evidence so far to demonstrate that the osteogenic differentiation of human MSCs induced by PEMFs involves the activation of proteins belonging to the BMP signaling pathway confirming recent results on p38 MAPK reported in rat calvarial osteoblasts [35]. On the other hand, the activation of MAPKs induced by EMFs was reported in other cell models [36,37]. In addition, the pattern of protein activation in the presence of both BMP2 and PEMFs suggests that the osteogenic effects observed when the two stimuli are used in combination may be due to the contemporary augmented activation of both SMAD1/5/8 and p38 MAPK. 

Finally, to further confirm the involvement of SMAD1/5/8 and p38 MAPK activation in hMSCs differentiation induced by BMP2, PEMFs, or both, we also evaluated the impact of DM, a small BMP signaling inhibitor [22,23,24] and of SB203580, a commonly used p38 MAPK inhibitor on the osteogenic parameters [25]. In all our experimental conditions, DM treatment used at a dose able to significantly inhibit SMAD1/5/8 activation without affecting cell viability, almost completely abrogated cell differentiation, by inhibiting expression of DLX5 and RUNX2 osteogenic transcription factors, ALP activity, and OC production. Notably, these data designate for the first time that SMAD 1/5/8 activation plays a crucial role, not only in BMP2-induced differentiation, but also PEMF-induced hMSCs osteogenic differentiation [17,18]. Results obtained in the presence of SB203580 showed a significant impact of p38 inhibition on the osteogenic parameters investigated. Specifically p38 inhibition did not modify DLX5 gene expression, whilst it reduced the other osteogenic parameters investigated although globally with a minor effect compared to SMAD 1/5/8 inhibition. In addition, these data showed the relevance of the MAPK signaling pathways, and specifically p38 pathways, as major regulators of osteogenic differentiation and in bone cells response to a multiplicity of signals including hormones and growth factors, extracellular matrix binding and physical forces [25,38].

## 4. Materials and Methods 

### 4.1. Cell Cultures

hMSCs from bone marrow were purchased from Lonza (Lonza, Walkersville, MD, USA), grown in T75 culture flasks (Falcon BD, Franklin Lakes, NJ, USA) and incubated in standard conditions (37 °C, 5% CO_2_). Cells were cultured in complete mesenchymal stem cell basal medium (MSCBM) (Lonza) and used at the third passage for the later differentiation experiments.

### 4.2. Osteogenic Differentiation and Cell Treatments 

Cells were seeded at the concentration of 4000 cells/cm^2^ in multi-well plates for ALP, OC, alizarin red staining, real time-PCR, and Western blot assays. To induce differentiation, cells were cultured in OM containing dexamethasone, L-glutamine, ascorbate, penicillin/streptomycin, β-glycerophosphate, and growth factors (Lonza) [7,8]. Cultures were randomly assigned to the following groups:

- OM + BMP2;
- OM + PEMFs;
- OM + PEMFs + BMP2.

For BMP2 treatments recombinant Human/Murine/Rat BMP2 (CHO derived) (PeproTech EC, Ltd. London) was used. In preliminary experiments we evaluated the effects of increasing doses of BMP2 (0, 1, 10, 50, 100 ng/mL) on osteogenic markers, including OC and mineralization, up to 28 days [7,8]. The medium was changed twice a week. Osteogenic markers and gene expression by real-time PCR and Western blotting were analyzed at several experimental endpoints (3, 7, 14, 21, and 28 days). Control cultures were maintained in MSCBM (Lonza, Walkersville, MD, USA).

### 4.3. PEMFs and Exposure Conditions

As in previous studies [5,7,39,40,41], PEMFs with specific biophysical characteristics were applied to stimulate osteogenic differentiation of hMSCs. PEMF were delivered by a pulse generator (IGEA, Carpi, Italy), previously described [7,8]. The specific characteristics of the signal were 1.5 mT intensity, 1.3 ms pulse duration, and 75 Hz frequency. PEMFs were administered for the whole differentiation time (28 days) according to previous studies [7,8].

### 4.4. SMAD and MAPK Signaling Inhibition

Dorsomorphin (DM (6-[4-(2-piperidin-1-yl-ethoxy)phenyl]-3-pyridin-4-yl-pyrazolo[1,5-a]pyrimidine) (Sigma-Aldrich S.r.l., Milano, Italy), a selective inhibitor of BMP type I receptors [22,23,24] and SB203580 (Cell Signaling Technology Inc., Euroclone, Milano, Italy), a pyridinyl imidazole inhibitor widely used to inhibit p38 MAPK [25], were added to culture medium in the three treatment groups (OM + PEMFs; OM + BMP2; OM + PEMFs + BMP2) for the whole culture period. SB203580 was used at 10 µM as commonly recommended for cell culture experiments [25,42], DM was used at different concentrations (0, 5, 10, 20 μM) [24] in preliminary experiments to evaluate p-SMAD1/5/8 activation by Western blotting. Cell viability was assessed using Prestoblue assay (Invitrogen by Life Technologies, Monza, Italy) [41].

### 4.5. Osteogenic Markers

#### 4.5.1. Alkaline Phosphatase (ALP) Activity

ALP activity was spectrophotometrically evaluated as previously reported in our studies [7,8]. Briefly, after a washing in PBS, cells were lysed by 0.1% Triton X 100 (Sigma-Aldrich) and incubated at 37 °C for 30 min with 10 mM p-nitrophenylphosphate (p-NP) (Sigma-Aldrich) in alkaline buffer (100 Mmdiethanolamine and 0.5 mM MgCl2, pH 10.5). The reaction was blocked with 0.2 M NaOH and the absorbance (at 405 nm) measured by Jenway 6305 Spectrophotometer (Barloworld Scientific, Dunmow, Essex, UK). ALP activity was normalized to total deoxyribonucleic acid (DNA) content and expressed in μM/(min × μg DNA).

#### 4.5.2. Osteocalcin (OC) Levels

OC levels were detected as previously described [7,8], using a commercially available ELISA kit (Invitrogen, Rockville, MD, USA) in which monoclonal antibodies are directed against human OC distinct epitopes. OC levels were expressed in ng/µg DNA.

### 4.6. Alizarin Red S (ARS) Staining and Quantification

To evaluate mineralization, cell cultures were stained with alizarin red according to previous studies [43]. In brief, the monolayers were stained by fixing calcium deposits in 10% formaldehyde followed by incubation with 2% Alizarin Red S (Histo-Line Laboratories S.r.l, Milano, Italy) for 30 min at room temperature. Images were captured using a standard light microscope (Nikon Eclipse TE 2000-E microscope, Nikon Instruments Spa, Sesto Fiorentino, FI, Italy) equipped with a digital camera (DXM 1200F; Nikon Instruments Spa, Italy). Further, the mineralization was quantified dissolving alizarin red stain with a solution of 20% methanol and 10% acetic acid (Sigma-Aldrich) for 15 min. The reading at 450 nm was carried out using the Jenway 6305 spectrophotometer (Barloworld Scientific) [8].

### 4.7. Quantitative Real-Time PCR (qPCR)

Total RNA was isolated from hMSCs using the PureLink RNA minikit (Invitrogen by Life Technologies) according to the manufacturer’s specifications. Following treatment with DNAse, RNA concentration and purity were determined by NanoDrop 2000 spectrophotometer (Thermo Scientific, Inc., MA, USA). Real-time PCR was performed according to previous studies [8,43]. Briefly, 2 µg of total RNA were reverse transcribed to cDNA using theSuperScript™ III First-Strand Synthesis system for RT-PCR (Invitrogen by Life Technologies). cDNA mixture was amplified using PerfeCta SYBR Green SuperMix ROX kit (Quanta Biosciences by VWR, Milano, Italy) according to the producer’s instructions in a final volume of 20μL. Primers were used at 500 nM concentration (Sigma-Aldrich) [8,44,45]. Real-time PCR was carried out for DLX5, RUNX2, BMP2, BMP6, BMP9, Type I BMP receptors (ALK2/ACVR1, ALK3/BMPR-IA, ALK6/BMPR-IB) and Type II BMP receptors (BMPR-II) in a 7500 Fast Real-Time PCR system (Applied Biosystems, Life Technologies, Waltham, MA, USA).

All reactions were performed in triplicate and gene expression was assessed by using the 2^−ΔΔCt^ method. Gene expression levels of the target genes were calculated by normalization to the reference gene glucuronidase beta (GUSB) [8], using control cells, as calibrators.

### 4.8. Western Blotting and Densitometric Analysis

Western blot analysis was performed to identify SMAD1/5/8, p38 MAPK, and ERK1/2 MAPK proteins activation in all the tested experimental conditions at 4 and 24 h. Cell monolayers were treated with buffer solution (PBS, 0.1% SDS, 1% NP40, 0.5% sodium deoxycolate, 1 mM PMSF, 10 μg/mL pepstatin, 10 μg/mL leupeptin, 1 mM sodium orthovanadate, 10 mM NaF and 10 mM β-glycerophosphate) for 1 h at 4 °C. Protein samples were subjected to SDS-PAGE and immunoblotting [8,41]. Membranes were incubated with the following antibodies: p-SMAD1/5/8 Ser465/467 and p-p38 Thr180/Tyr182 and Phospho-p44-42 MAPK (ERK1/2) (Thr202/204) (Cell Signaling Technology Inc.). β-actin (Sigma-Aldrich) was used as control.

Membranes were incubated overnight at 4 °C with the primary antibody, washed in tris-buffered saline/Tween, and subsequently incubated for 1 h at room temperature with the secondary antibody peroxidase-conjugated in tris-buffered saline/Tween containing 5% nonfat dry milk.

Membranes were developed using ECL Western Blotting Detection Reagents (Life Technologies, Carlsbad, CA, USA). Protein immunoreactive bands were analyzed and quantitated by using Image Lab software 4.0 (Bio-Rad, Hercules, CA, USA). Data obtained were as the ratio of each band density to the respective β-actin band density.

### 4.9. Statistical Analysis

Statistical evaluation of data was performed using two-way ANOVA followed by Tukey’sposthoc test used for multiple comparison. Data were reported as means ± standard deviations. For each experiment, experimental condition was tested in triplicate. The *p*-value ≤ 0.05 was considered statistically significant. The analysis was carried out using the GraphPad Prism version 6.01 Software (GraphPad Software, San Diego, CA, USA).

## 5. Conclusions

Our results confirm the combined activity of biophysical stimulation with PEMFs and BMP2 for a more efficient osteogenesis of human bone marrow MSCs. Further, they suggest that PEMFs could be a positive stimulator of the BMP signaling pathway. Specifically, our results identify BMP signaling components as molecular targets involved in PEMFs induced osteogenic differentiation and the ability of PEMFs to enhance BMP2 activity in hMSCs. Notably, in the present study we show that SMAD1/5/8 activation is permissive to the osteogenic differentiation induced by PEMFs. Further we show the relevance of the MAPK signaling pathway, and specifically the involvement of p38 activation. Collectively, the cumulative effects of BMP2 and PEMFs indicate a complex interplay between different signaling pathways in driving osteogenic differentiation of hMSCs. In conclusion, our data add new information concerning the molecular mechanism by which PEMFs may modulate cell behavior [8,40]. Due to the use of both PEMFs and BMP2 treatment in clinics, studies in animals and further knowledge about such interaction between PEMFs and BMP signaling could be extremely important as in vivo administered rhBMP2 has shown clinical side-effects requiring strategies to improve efficacy by using lower doses.

## Abbreviations

PEMFs	Pulsed electromagnetic fields
MSCs	Mesenchymal stem cells
BMP2	Bone morphogenetic protein-2
hMSCs	Human MSCs
EMF	Electromagnetic field
BMPs	Bone morphogenetic proteins
TGF-β	Transforming growth factors beta
CHO	Chinese hamster ovary
rhBMP2	Recombinant human BMP-2
MAPKs	Mitogen-activated protein kinases
OSX	Osterix
DLX	Distal-less homeobox
MSCBM	Mesenchymal stem cell basal medium
ALP	Alkaline phosphatase
OC	Osteocalcin
OM	Osteogenic medium
DM	Dorsomorphin
p-NP	p-nitrophenylphosphate
DNA	Deoxyribonucleic acid
ARS	Alizarin red Staining
qPCR	Quantitative PCR
GUSB	Glucuronidase beta
